# ABCA7 Risk Genotype Diminishes the Neuroprotective Value of Aerobic Fitness in Healthy Older African Americans

**DOI:** 10.3389/fnagi.2019.00073

**Published:** 2019-04-09

**Authors:** Chelsie N. Berg, Neha Sinha, Mark A. Gluck

**Affiliations:** Center for Molecular and Behavioral Neuroscience, Rutgers University-Newark, Newark, NJ, United States

**Keywords:** ABCA7, African American, Alzheimer’s disease, aerobic fitness (VO_2_ max), cognitive decline

## Abstract

Although the association of ABCA7 risk variants with Alzheimer’s disease (AD) has been established worldwide, its effect size on the relative odds of being diagnosed with AD is significantly higher in African Americans. Across ethnicities, two common ABCA7 loci (rs115550680 and rs3764650) have been confirmed to increase the risk of AD. While ABCA7 rs115550680 has been linked to the development of late-onset AD in African Americans, no association between ABCA7 variant rs3764650 and AD has been found in this population. In order to elucidate the influence of ABCA7 rs3764650 on AD risk in African Americans, we sought to investigate the relationship between this variant, aerobic fitness, and cognition. The present study tested the hypothesis that in African Americans, ABCA7 rs3764650 confers an indirect risk for AD via its interaction with aerobic fitness, a modifiable lifestyle factor known to attenuate AD-related neuropathology. In a case-control sample of 100 healthy African Americans, we observed that ABCA7 rs3764650 genotype modulates the association between aerobic fitness and a cognitive assessment of generalization following rule learning. For carriers of the non-risk genotype, higher levels of aerobic fitness were significantly associated with fewer generalization errors, while carriers of the risk genotype did not show any relationship between aerobic fitness and generalization. Our findings imply that ABCA7 rs3764650 risk genotype may diminish the neuroprotective effects of aerobic fitness, and, they suggest differing risk patterns between cognitive decline and fitness by ABCA7 genotype. Thus, in African Americans the interactive effects of ABCA7 rs3764650 and aerobic fitness likely compound overall ABCA7-related AD risk, and may contribute to health disparities whereby African Americans are at a higher risk for dementia, with double the prevalence of AD.

## Introduction

African Americans are at a heightened risk for age-related cognitive decline and memory loss, with twice the prevalence of Alzheimer’s disease (AD) compared to Caucasian Americans (Tang et al., 2001; Barnes and Bennett, 2014; Alzheimer’s Association, 2018). The underlying causes of this health disparity are not sufficiently understood. Previous research has found that APOE ε4 genotype is associated with 2–3 times the risk of AD in heterozygotes and 12 times the risk in homozygotes (Michaelson, 2014). Outside of APOE ε4, ABCA7 is one of the strongest genetic predictors of AD in African Americans (Reitz et al., 2013). Additionally, modifiable lifestyle factors such as diet, exercise and aerobic fitness, contribute to AD risk. Aerobic fitness is associated with decreased cognitive decline and reduced risk of AD (Colcombe and Kramer, 2003; Kramer et al., 2005, 2006). Specifically in the medial temporal lobe (MTL), one of the earliest brain regions impacted by the disease process, the hippocampus is a major site of neuroplasticity that is sensitive to the effects of physical activity (Cotman et al., 2007). Increased aerobic training has been found to increase hippocampal volume and cerebral blood flow (Burdette et al., 2010; Erickson et al., 2011), suggesting that aerobic fitness may have neuroprotective effects on hippocampal structure and function. Recent work also indicates that increased levels of aerobic fitness can diminish the negative effects of risk genes involved in lipid metabolism; the combined adverse influence of polygenic risk derived from APOE ε4, CLU, and ABCA7, on AD biomarkers, was lessened in those with higher levels of cardiovascular fitness (Schultz et al., 2017).

As noted above, ABCA7 is one of the strongest genetic risk factors of AD in African Americans (Reitz et al., 2013). ABCA7 is a member of the superfamily of ATP-binding cassette (ABC) transporters which function to regulate the homeostasis of phospholipids and cholesterol in the central nervous system and peripheral tissues. It is expressed in a variety of tissues/organs, including the brain, as well as, blood cells. Accumulating evidence through genetic studies suggests that the contribution of ABCA7 to AD risk is mediated by the dysfunction of ABCA7 expression (Aikawa et al., 2018), such that, increased ABCA7 expression levels have been associated with more severe cognitive deficits in AD subjects (Karch et al., 2012). In particular, the ABCA7 single nucleotide polymorphism (SNP) rs3764650 has been implicated in influencing ABCA7 expression levels in the brain (Vasquez et al., 2013), and corresponds to ~10%–20% increased risk of AD in Caucasians (Hollingworth et al., 2011; Naj et al., 2011). This ABCA7 variant is associated with a later age of onset and shorter disease process (Karch et al., 2012; Zhao et al., 2015), exacerbating cognitive decline in subjects diagnosed with mild cognitive impairment or AD (Carrasquillo et al., 2015).

An association between ABCA7 SNP rs3764650 and AD has not been found in GWAS (genome-wide association studies) in African American cohorts. Another variant, ABCA7 rs115550680, has been linked to the development of late-onset AD in African Americans (Reitz et al., 2013). We have previously demonstrated that in non-demented African American elderly, rs115550680 corresponds to impairments in MTL network function commensurate with hippocampus-related cognitive deficits (Sinha et al., 2018). Despite ABCA7 rs3764650 not being directly implicated in AD in African Americans, we hypothesized that it may yield an indirect risk through its interaction with other risk factors, which in turn, could account for the higher incidence rate of dementia and AD in this population.

Studies examining the interaction between APOE ε4 and physical fitness have revealed a moderating effect of AD genetic risk on the association between physical activity and cognitive function (Schuit et al., 2001; Podewils et al., 2005; Smith et al., 2011). Furthermore, considering the recent finding that aerobic fitness attenuates the adverse influence of AD-related polygenic vulnerability derived from genes implicated in lipid homeostasis, including ABCA7 (Schultz et al., 2017), we sought to investigate if ABCA7 rs3764650 confers AD risk in African Americans by moderating the neuro protective effects of aerobic fitness.

The risk allele for rs3764650 (G) is related to increased hippocampal atrophy (Ramirez et al., 2016), whereas aerobic fitness is linked to increased neurogenesis in the hippocampus (Van Praag et al., 2005; Van Praag, 2008). We measured hippocampus-related cognitive function through performance on the concurrent discrimination and generalization task (Myers et al., 2002). During this two-phased task, participants learn a series of visual discriminations and then have their generalization abilities tested after the stimulus information changes. This task selectively engages the hippocampus (Johnson et al., 2008), and, unlike other standard cognitive tests, performance can differentiate hippocampal-atrophied from non-atrophied individuals (Myers et al., 2002, 2008). Using this task, the present study investigated the relationship between ABCA7 rs3764650 genotype and aerobic fitness, and their combined influence on hippocampal function and potential AD risk, in a group of healthy older African Americans who were either carriers of the non-risk (TT) or high-risk (GG) genotypes.

## Materials and Methods

### Participants

Participants in this study were recruited through longstanding partnerships with local churches; senior centers; city, county, and state offices for health and aging; as well as from outreach to public housing and other federally-subsidized low-income housing sites. For additional details on our community engagement, outreach, and recruitment strategies, see www.brainhealth.rutgers.edu as well as Gluck et al. (2018).

One-hundred individuals participated in our present study with a case-control matched design. Fifty individuals were homozygous for the ABCA7 rs3764650 non-risk “T” allele. We then matched these non-risk individuals with participants who were homozygous for the risk “G” allele, based on age and years of education. Overall, the current study included 20 males (14 in the non-risk TT group and six in the risk GG group) and 80 females, aged 55–86 years, with an average age of 67 years (Table 1).

**Table 1:** Demographics and neuropsychological tests.

Measure	ABCA7 rs3764650 non-risk group (TT)	ABCA7 rs3764650 risk group (GG)	Difference
Sample size	50	50	
Age	66.54 (5.643)	67.12 (6.826)	p = 0.644
Education	13.76 (2.50)	13.96 (3.055)	p = 0.721
BMI	30.72 (6.816)	30.07 (6.465)	p = 0.625
MMSE	27.98 (1.785)	27.38 (2.258)	p = 0.146
RAVLT (Delayed Recall)	7.40 (3.175)	6.70 (3.581)	p = 0.304
WAIS-IV Digit Span	22.62 (4.615)	22.24 (5.487)	p = 0.709
NAART	39.90 (10.691)	36.7 (12.154)	p = 0.165
BDI	7.82 (4.939)	7.56 (7.770)	p = 0.842

Those who identified as African American and were at least 55 years old were eligible for participation. However, certain medical diagnoses, medications, and lifestyle factors can impact cognitive function to a level that may confound study results; therefore, to limit our findings to healthy older adults, participants exhibiting signs of dementia, evident from the standardized neuropsychological assessments (below) or who took medications that can affect cognition were excluded from the study. Other exclusion criteria included: excessive alcohol and/or drug use, psychiatric disorders (such as Bipolar Disease and Schizophrenia), seizure disorders (such as Epilepsy), and significant cerebrovascular or cardiovascular diseases. All subjects gave written informed consent in accordance with the Declaration of Helsinki. The protocol was approved by the Rutgers University-Newark Institutional Review Board.

### Standardized Neuropsychological Assessments and Self-Report Measures

In order to assess cognition and exclude anyone showing signs of cognitive impairment consistent with early dementia or other age-related disorders, we gave participants a battery of neuropsychological tests. The neuropsychological battery consisting of the Mini Mental State Exam (MMSE; broad assay of cognitive impairment), Rey Auditory Verbal Learning Test (RAVLT) Delayed Recall (verbal memory), North American Adult Reading Test (NAART35; verbal intellectual ability), and Wechsler Adult Intelligence Scale (WAIS-IV) Digit Span (working memory) was administered (Table 1). The Beck Depression Inventory (BDI) was also administered to measure characteristic attitudes and symptoms of depression.

### Aerobic Fitness Assessment

In addition to measuring height and weight, the Six minute Walk was used to characterize aerobic fitness. Participants were instructed to walk a premeasured length on a flat surface for 6 minutes, with the goal of covering as much ground as possible (McGavin et al., 1976, 1978). At the completion of the 6 minutes, total walking distance was recorded in meters (Noonan and Dean, 2000). To approximate participants’ maximal oxygen consumption, we utilized the equation determined by Ross et al. (2010): VO_2_ max = [4.948 + (0.023 * Distance)]. This measure of maximal oxygen consumption (VO_2_ max) is widely recognized as both a representation of the functional limitations of the cardiovascular system as well as a measure of aerobic fitness (Taylor et al., 1995).

### Behavioral Paradigm: Concurrent Discrimination and Generalization Task

Testing took place in a quiet room, with the participant seated in front of a laptop computer with a color screen. The keyboard was masked except for two keys, labeled “Left” and “Right, ” which the participant used to enter responses.

**Figure 1:**
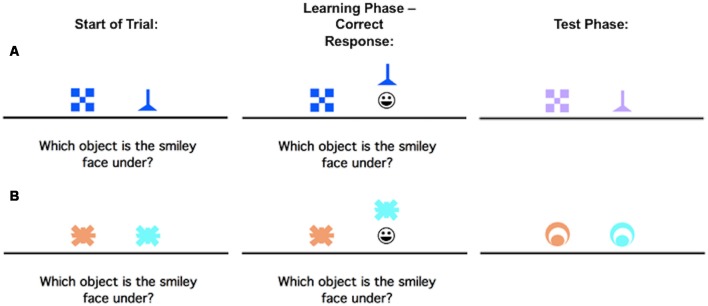
An example of the concurrent discrimination and generalization task. On each trial of Phase 1 (acquisition/learning), the discrimination pair is presented and if the participant responds correctly, the chosen object is raised to reveal a smiley face icon underneath. During Phase 2 (generalization/test), events are similar to Phase 1, but the objects are changed so that the relevant feature remains the same, but the irrelevant feature is novel. **(A)** is an example of a trial where the relevant feature is shape, but not color, while **(B)** is an example of a trial where the relevant feature is color, but not shape.

The concurrent discrimination and generalization task has been previously described in Myers et al. (2002), but to summarize, it is a two-phase task in which participants learn a series of visual discriminations and are then tested on their ability to generalize when the stimulus information changes. Phase 1 (acquisition) involves an 8-pair concurrent discrimination. For each trial, two colored shapes appeared, approximately 1-inch high on the screen and set about 3 inches apart (approximately 1.5° of visual angle, at normal viewing distance). The participants were instructed to press the “Left” or “Right” key to choose one object. Onscreen, the chosen object rose and if the choice was correct, a smiley face was revealed underneath (Figure 1). There was no limit on response time, but there was an interval of about 1 s between participant response and start of the next trial, allowing the participant to view the discrimination pair and feedback (presence or absence of the desired smiley face icon).

No information about the correct object was given ahead of time, making this an incrementally acquired, feedback-based learning task in which participants had to learn which object was correct. Within each object pair, the same object was always rewarded. For four of the discrimination pairs, objects differed in shape but not color (example, blue checkerboard vs. blue funnel); for the other four pairs, objects differed in color but not shape (example, orange spider vs. blue spider). Thus, within each pair, one dimension (shape or color) was relevant to predicting the location of the smiley face, and one dimension was irrelevant. Trials were organized into blocks, each containing 16 trials: one presentation of each discrimination pair in both possible left-right ordering. Trials in a block occurred in a pseudo-random but fixed order. Phase 1 continued until the participant reached a criterion of 16 consecutive correct responses, or for a maximum of 96 trials (six blocks).

As soon as the acquisition phase ended, phase 2 (generalization) began without any warning to the participant. The screen events were identical to the concurrent discrimination phase except that the discrimination pairs were altered so that the relevant features remained constant while the irrelevant features were altered. For example, the blue checkerboard vs. blue funnel might change to lavender checkerboard vs. lavender funnel as shown in the first row, second column (Figure 1A); the shapes remain the same but the irrelevant color changes from blue to lavender. In the second example, the orange vs. blue spider discrimination might change to an orange vs. blue circle (Figure 1B); the shape remains irrelevant, but the color continues to be predictive.

Individuals who had solved the concurrent discrimination by basing associations on the relevant features (funnel beats checkerboard and blue beats orange) could perform perfectly in the generalization phase since the relevant features were still predictive. By contrast, individuals who had approached the concurrent discrimination phase by learning to respond to whole objects (blue funnel beats blue checkerboard), by treating all features equally, are effectively confronted with novel objects (lavender funnel and lavender checkerboard) in the generalization phase and might perform near chance.

The generalization phase was organized into blocks of 16 trials, one trial with each discrimination pair in both possible left-right ordering, in a pseudo-random but fixed order. It continued until the participant reached a criterion of 16 consecutive correct responses or a maximum of 96 trials (six blocks). The entire procedure took about 15–20 min to complete.

## Results

All participants underwent a battery of standardized neuropsychological assessments and were included in our analyses only if they were within the age and education-adjusted norms (Table 1). No differences were observed on the standardized measures of cognitive functioning (MMSE, Digit Span, NAART, RAVLT) or depression level (BDI).

**Figure 2:**
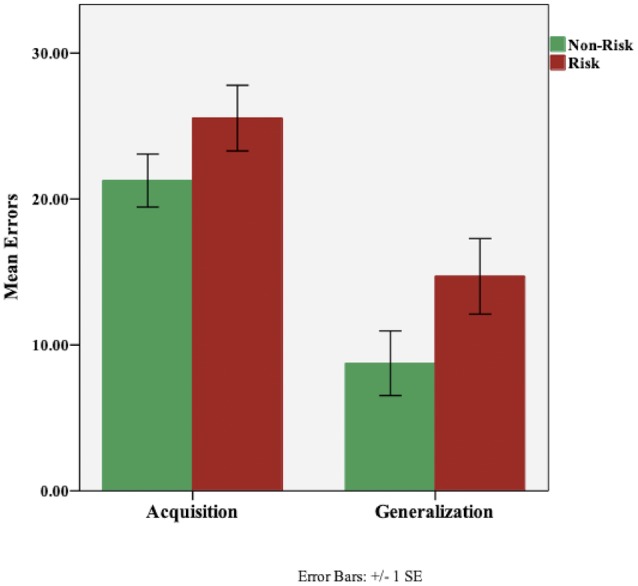
Performance (total errors) on the concurrent discrimination and generalization task based on ABCA7 genotype: Non-Risk (TT) vs. Risk (GG). There were no group differences based on ABCA7 genotype on initial learning (acquisition; p = 0.159) or generalization (p = 0.287). Although, the risk group made more generalization errors, the effect of group was not significant.

On the concurrent discrimination task, all participants reached the criterion of 16 consecutive correct responses on the acquisition phase, indicating that they successfully learned the task. Figure 2 shows the mean errors for the acquisition and generalization phases of the task. A one-way analysis of covariance (ANCOVA) was performed with ABCA7 genotype as the fixed factor, and, Digit Span total score, NAART total errors, and RAVTL-delayed recall scores as covariates. There was no effect of group, based on ABCA7 genotype (F(1,95) = 2.01, p = 0.159), on acquisition. Among the covariates, the total score on the Digit Span task was a significant predictor of acquisition errors (F(1,95) = 8.01, p = 0.006). In order to look at ABCA7-related differences in generalization scores, acquisition errors were entered as an additional covariate in the ANCOVA. While the risk group made more generalization errors (M = 14.7, SD = 18.3) than the non-risk group (M = 8.74, SD = 15.7), the effect of group was not significant (F(1,94) = 1.144, p = 0.287); there was however a significant effect of acquisition errors (F(1,92) = 146.21, p = 0.001) and trending effect of RAVLT-delayed recall (F(1,92) = 3.79, p = 0.055).

**Figure 3:**
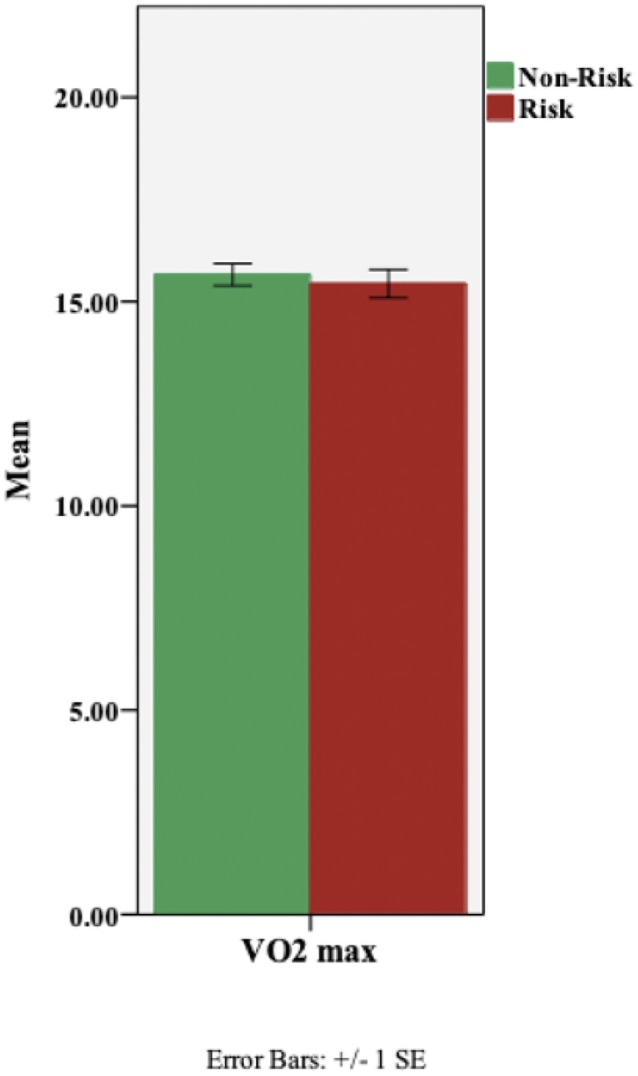
Aerobic fitness (VO_2_ max) based on ABCA7 genotype: Non-Risk (TT) vs. Risk (GG). There was no effect of genotypic group on VO_2_ max (p = 0.561).

Using height and weight measures, the participants’ body mass index (BMI) was computed. No ABCA7-related differences were found in BMI (Table 1). Next, we assessed the effect of ABCA7 genotype on aerobic fitness (VO_2_ max); a one-way ANCOVA was performed with ABCA7 genotype as the fixed factor, and, BMI as a covariate. There was no effect of group, based on ABCA7 genotype (F(1,97) = 0.34, p = 0.561), on aerobic fitness (Figure 3).

**Figure 4:**
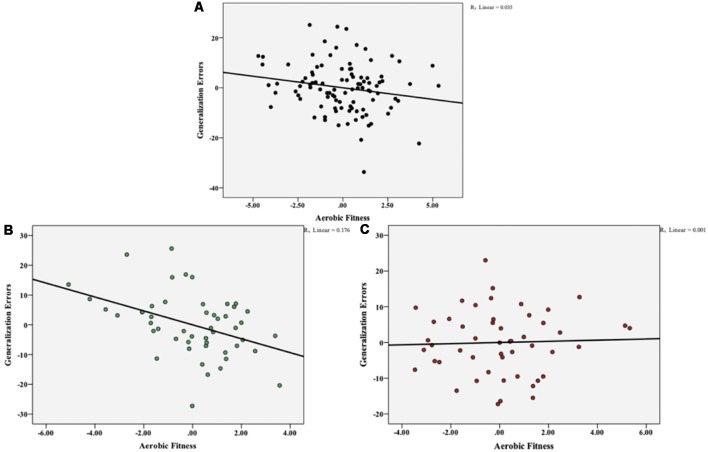
Overall aerobic fitness (VO_2_ max) was negatively correlated with total generalization errors, but this relationship did not reach significance (p = 0.071) **(A)**. At the individual group level, there was a significant negative correlation between VO_2_ max and generalization errors for the non-risk group (p = 0.004) **(B)**, but not for the risk group (p = 0.809) **(C)**.

**Figure 5:**
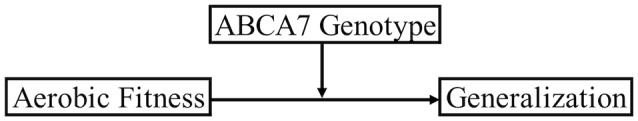
ABCA7 genotype significantly moderates the relationship between aerobic fitness and generalization.

To investigate the potential link between aerobic fitness and the ability to generalize, we performed partial correlations between VO_2_ max and generalization total errors, controlling for the effect of acquisition errors, BMI, and performance on standardized neuropsychological assessments (Digit Span, NAART, RAVLT). Aerobic fitness was negatively correlated with generalization errors (r(93) = −0.186, p = 0.071), but this relationship did not reach significance (Figure 4A). In order to determine the effect of ABCA7 genotype on this negative association between aerobic fitness and generalization, we computed the correlations at the individual group level. As shown in Figure 4B, there was a significant negative correlation between VO_2_ max and generalization errors for the non-risk group (r(43) = −0.419, p = 0.004), but not for the risk group (r(43) = 0.037, p = 0.809; Figure 4C). Furthermore, a hierarchical linear regression (HLR) revealed that when controlling for acquisition errors, BMI, and standardized neuropsychological assessment scores, ABCA7 genotype significantly moderates the relationship between aerobic fitness and generalization (R^2^ change = 0.066, F(1,91) = 23.85, p = 0.001; Figure 5).

## Discussion

In this study, we observed that ABCA7 rs3764650 genotype modulates the association between aerobic fitness and generalization. For carriers of the non-risk genotype, higher levels of aerobic fitness were significantly associated with fewer generalization errors, while carriers of the risk genotype did not show any relationship between aerobic fitness and generalization. Importantly, there were no group differences in either aerobic fitness or generalization performance based on ABCA7 rs3764650 status.

Healthy elderly with higher fitness levels show less cognitive decline (Yaffe et al., 2001; Barnes et al., 2003) and reduced risk for dementia (Podewils et al., 2005; Larson et al., 2006) than those with lower fitness levels. Attenuation of atrophy in the MTL, one of the earliest loci of AD, is also associated with greater cardiorespiratory fitness in AD patients (Honea et al., 2009). In particular, the hippocampus is one of the major brain sites of neuroplasticity, and several studies have demonstrated marked benefits of aerobic fitness and physical activity on hippocampal structure and related cognitive function (Pereira et al., 2007; Erickson et al., 2009, 2011; Bugg and Head, 2011; Szabo et al., 2011; Maass et al., 2015; Rehfeld et al., 2017). In the present study, the benefit of aerobic fitness on hippocampal function, as measured by performance on the concurrent discrimination and generalization task, was observed only in carriers of the ABCA7 protective TT genotype, but not in carriers of the risk GG genotype. Our results, therefore, indicate that ABCA7 risk genotype may attenuate the neuroprotective value of aerobic fitness in cognitively healthy older adults. To our knowledge, this is the first study to demonstrate the interactive effect of an ABCA7 variant (rs3764650) and aerobic fitness on hippocampus-related cognitive functioning.

There were no significant variations in aerobic fitness between the risk GG and non-risk TT genotypes, indicating that ABCA7 rs3764650 does not directly affect aerobic fitness. However, the significant interaction between ABCA7 genotype and aerobic fitness indicates that both may be impacting common causal factors involved in hippocampal dysfunction. The results of our study, therefore, suggest an effect of aerobic fitness on hippocampal function through ABCA7-related mechanisms. Studies to date have implicated two possible mechanisms whereby ABCA7 rs3764650 contributes to AD pathology, both of which, in turn, result in hippocampal neurodegeneration. First, it increases amyloid deposition (Shulman et al., 2013; Ma et al., 2018), thereby initiating hippocampal dysfunction via reciprocal connections through the entorhinal cortex (Reitz et al., 2009; Pooler et al., 2015). Second, it interferes with the transportation of brain lipids (Giri et al., 2016; Li et al., 2017), resulting in dyslipidemia (abnormal lipid levels) which induces structural damage to the hippocampus (Zhao et al., 2017). Moreover, aerobic activity has been implicated in aiding brain lipid homeostasis (Houdebine et al., 2017), as well as, reducing the accumulation of Aβ deposits (Maesako et al., 2012; He et al., 2017). It, therefore, seems likely that ABCA7 rs3764650 risk genotype negates the influence of aerobic fitness on one or both these mechanisms, and in turn, reduces any subsequent neuroprotective effects on the hippocampus, thereby resulting in increased risk for AD.

Although the association of ABCA7 rs3764650 with AD has been confirmed in Caucasians (Harold et al., 2009; Lambert et al., 2010; Carrasquillo et al., 2011; Hollingworth et al., 2011; Omoumi et al., 2014; Cuyvers et al., 2015), GWAS studies in African Americans have found either none, or a nominally significant association (Logue et al., 2011; Reitz et al., 2013; N’songo et al., 2017). It is possible that in African Americans, ABCA7 rs3764650 is not a causative variant. Consistent with this, in our current sample of cognitively healthy older African Americans, we did not see any difference in generalization errors or performance on the standardized neuropsychological assessments between carriers of the risk vs. non-risk genotype. Furthermore, in a previous study we investigated the effect of ABCA7 rs115550680, a variant with a significantly increased risk for AD exclusively in African-Americans (Reitz et al., 2013), on the same behavioral paradigm; we found that non-demented African American elderly with the ABCA7 rs115550680 risk genotype had impairments in generalization, mediated by cortico-hippocampal network dysfunction (Sinha et al., 2018). Taken together, these results indicate that in African Americans, ABCA7 rs3764650 does not confer a direct AD risk, but rather indirectly increases the risk of AD by diminishing the benefits of aerobic fitness. Hence, changes in fitness and perhaps other modifiable lifestyle factors may not ameliorate AD-related neurodegeneration, which contributes to health disparities whereby African Americans are at a higher risk for dementia, with double the prevalence of AD (Tang et al., 2001; Barnes and Bennett, 2014; Alzheimer’s Association, 2018).

Consistent with our results, Podewils et al. (2005) found that APOE ε4 carriers did not attain the same benefit as non-carriers from physical activity, such that, physical activity reduced the risk for dementia only in non-carriers of the ε4 allele. Other studies, however, have yielded contrary findings, reporting that the protective effects of physical activity on future cognitive decline was specific to APOE ε4 carriers (Schuit et al., 2001; Rovio et al., 2005; Smith et al., 2011). These other studies used self-reported measures of physical activity and investigated the effect of APOE in Caucasian cohorts, which are notable differences from our study; nonetheless, with the exception of Podewils et al. (2005), these results are in contrast to those found in the current study. In our sample, we did not find either a direct effect of APOE ε4 status or an interaction between ABCA7 genotype and ε4 status. However, the small number of APOE ε4 carriers in our sample limits any conclusions. Future studies with a larger sample size are warranted to explore racial differences in the effects of APOE ε4 and potential gene-gene interactions.

Another unexplored limitation of the current study involves the imbalanced sex breakdown, with 20 males and 80 females between the two groups. Previous research in ABCA7 knockout mice found evidence of subtle sex differences though behavioral impairments in differing cognitive domains between males and females (Logge et al., 2012). However, in humans, sex differences in cognitive impairments associated with ABCA7 remain largely unexplored. Furthermore, GWAS studies exploring the role of ABCA7 did not find any sex-dependent differences in AD risk (Logue et al., 2011; Reitz et al., 2013; N’songo et al., 2017). In the future, studies with evenly distributed samples are required to answer any questions regarding sex differences in ABCA7-related risk for cognitive deficits and AD.

In conclusion, the results of this study show that in cognitively healthy elderly African Americans, ABCA7 risk variant rs3764650 moderates the relationship between aerobic fitness and hippocampal cognitive functioning. Our findings, therefore, suggest that carriers of the risk genotype are less likely to benefit from the disease-modifying effects of fitness, and any potential protective effect associated with aerobic fitness may not be enough to overcome AD-related neuropathology.

## Author Contributions

CB and NS performed statistical analyses, interpreted the data, and drafted the manuscript. MG designed the study and provided a critical review of the manuscript.

## Conflict of Interest Statement

The authors declare that the research was conducted in the absence of any commercial or financial relationships that could be construed as a potential conflict of interest.

## References

[B1] Aikawa T. Holm M.-L. Kanekiyo T. (2018). ABCA7 and pathogenic pathways of Alzheimer’s disease. Brain Sci. 8:27. 10.3390/brainsci8020027 29401741 PMC5836046

[B2] Alzheimer’s Association (2018). 2018 Alzheimer’s disease facts and figures. Alzheimer’s & Dementia 14, 367–429. 10.1016/j.jalz.2018.02.001

[B3] Barnes D. E. Yaffe K. Satariano W. A. Tager I. B. (2003). A longitudinal study of cardiorespiratory fitness and cognitive function in healthy older adults. J. Am. Geriatr. Soc. 51, 459–465. 10.1046/j.1532-5415.2003.51153.x 12657064

[B4] Barnes L. L. Bennett D. A. (2014). Alzheimer’s disease in african americans: risk factors and challenges for the future. Health Aff. 33, 580–586. 10.1377/hlthaff.2013.1353 24711318 PMC4084964

[B5] Bugg J. M. Head D. (2011). Exercise moderates age-related atrophy of the medial temporal lobe. Neurobiol. Aging 32, 506–514. 10.1016/j.neurobiolaging.2009.03.008 19386382 PMC2891908

[B6] Burdette J. Laurienti P. Espeland M. Morgan A. Telesford Q. Vechlekar C.. (2010). Using network science to evaluate exercise-associated brain changes in older adults. Front. Aging Neurosci. 2:23. 10.3389/fnagi.2010.00023 20589103 PMC2893375

[B7] Carrasquillo M. M. Belbin O. Hunter T. A. Ma L. Bisceglio G. D. Zou F.. (2011). Replication of EPHA1 and CD33 associations with late-onset Alzheimer’s disease: a multi-centre case-control study. Mol. Neurodegener. 6:54. 10.1186/1750-1326-6-54 21798052 PMC3157442

[B8] Carrasquillo M. M. Crook J. E. Pedraza O. Thomas C. S. Pankratz V. S. Allen M.. (2015). Late-onset alzheimer’s risk variants in memory decline, incident mild cognitive impairment and Alzheimer’s disease. Neurobiol. Aging 36, 60–67. 10.1016/j.neurobiolaging.2014.07.042 25189118 PMC4268433

[B9] Colcombe S. Kramer A. F. (2003). Fitness effects on the cognitive function of older adults: a meta-analytic study. Psychol. Sci. 14, 125–130. 10.1111/1467-9280.t01-1-01430 12661673

[B10] Cotman C. W. Berchtold N. C. Christie L.-A. (2007). Exercise builds brain health: key roles of growth factor cascades and inflammation. Trends Neurosci. 30, 464–472. 10.1016/j.tins.2007.06.011 17765329

[B11] Cuyvers E. De Roeck A. Van den Bossche T. Van Cauwenberghe C. Bettens K. Vermeulen S.. (2015). Mutations in ABCA7 in a belgian cohort of Alzheimer’s disease patients: a targeted resequencing study. Lancet Neurol. 14, 814–822. 10.1016/S1474-4422(15)00133-7 26141617

[B12] Erickson K. I. Prakash R. S. Voss M. W. Chaddock L. Hu L. Morris K. S.. (2009). Aerobic fitness is associated with hippocampal volume in elderly humans. Hippocampus 19, 1030–1039. 10.1002/hipo.20547 19123237 PMC3072565

[B13] Erickson K. I. Voss M. W. Prakash R. S. Basak C. Szabo A. Chaddock L.. (2011). Exercise training increases size of hippocampus and improves memory. Proc. Natl. Acad. Sci. U S A 108, 3017–3022. 10.1073/pnas.1015950108 21282661 PMC3041121

[B14] Giri M. Zhang M. Lü Y. (2016). Genes associated with Alzheimer’s disease: an overview and current status. Clin. Interv. Aging 11, 665–681. 10.2147/CIA.S105769 27274215 PMC4876682

[B15] Gluck M. A. Shaw A. Hill D. (2018). Recruiting older african americans to brain health and aging research through community engagement: lessons from the African-American Brain Health Initiative at Rutgers University-Newark. Generations 42, 78–82. Available online at: https://www.ingentaconnect.com/contentone/asag/gen/2018/00000042/00000002/art00013 30853750 PMC6404737

[B16] Harold D. Abraham R. Hollingworth P. Sims R. Gerrish A. Hamshere M. L.. (2009). Genome-wide association study identifies variants at CLU and PICALM associated with Alzheimer’s disease. Nat. Genet. 41, 1088–1093. 10.1038/ng.440 19734902 PMC2845877

[B17] He X. Liu D. Zhang Q. Liang F. Dai G. Zeng J.. (2017). Voluntary exercise promotes glymphatic clearance of amyloid beta and reduces the activation of astrocytes and microglia in aged mice. Front. Mol. Neurosci. 10:144. 10.3389/fnmol.2017.00144 28579942 PMC5437122

[B18] Hollingworth P. Harold D. Sims R. Gerrish A. Lambert J.-C. Carrasquillo M. M.. (2011). Common variants at ABCA7, MS4A6A/MS4A4E, EPHA1, CD33 and CD2AP are associated with Alzheimer’s disease. Nat. Genet. 43, 429–435. 10.1038/ng.803 21460840 PMC3084173

[B19] Honea R. Thomas G. P. Harsha A. Anderson H. S. Donnelly J. E. Brooks W. M.. (2009). Cardiorespiratory fitness and preserved medial temporal lobe volume in Alzheimer’s disease. Alzheimer Dis. Assoc. Disord. 23, 188–197. 10.1097/wad.0b013e31819cb8a2 19812458 PMC2760037

[B20] Houdebine L. Gallelli C. A. Rastelli M. Sampathkumar N. K. Grenier J. (2017). Effect of physical exercise on brain and lipid metabolism in mouse models of multiple sclerosis. Chem. Phys. Lipids 207, 127–134. 10.1016/j.chemphyslip.2017.06.002 28606714

[B21] Johnson S. C. Schmitz T. W. Asthana S. Gluck M. A. Myers C. (2008). Associative learning over trials activates the hippocampus in healthy elderly but not mild cognitive impairment. Aging Neuropsychol. Cogn. 15, 129–145. 10.1080/13825580601139444 17851984 PMC2645931

[B22] Karch C. M. Jeng A. T. Nowotny P. Cady J. Cruchaga C. Goate A. M. (2012). Expression of novel Alzheimer’s disease risk genes in control and Alzheimer’s disease brains. PLoS one 7:e50976. 10.1371/journal.pone.0050976 23226438 PMC3511432

[B23] Kramer A. F. Colcombe S. J. McAuley E. Scalf P. E. Erickson K. I. (2005). Fitness, aging and neurocognitive function. Neurobiol. Aging 26, 124–127. 10.1016/j.neurobiolaging.2005.09.009 16213062

[B24] Kramer A. F. Erickson K. I. Colcombe S. J. (2006). Exercise, cognition and the aging brain. J. Appl. Physiol. 101, 1237–1242. 10.1152/japplphysiol.00500.2006 16778001

[B25] Lambert J.-C. Grenier-Boley B. Chouraki V. Heath S. Zelenika D. Fievet N.. (2010). Implication of the immune system in Alzheimer’s disease: evidence from genome-wide pathway analysis. J. Alzheimers Dis. 20, 1107–1118. 10.3233/JAD-2010-100018 20413860

[B26] Larson E. B. Wang L. Bowen J. D. McCormick W. C. Teri L. Crane P.. (2006). Exercise is associated with reduced risk for incident dementia among persons 65 years of age and older. Ann. Intern. Med. 144, 73–81. 10.1097/00008483-200607000-00008 16418406

[B27] Li H. Zhou J. Yue Z. Feng L. Luo Z. Chen S.. (2017). A complex association between ABCA7 genotypes and blood lipid levels in Southern Chinese Han patients of sporadic Alzheimer’s disease. J. Neurol. Sci. 382, 13–17. 10.1016/j.jns.2017.09.016 29111006

[B28] Logge W. Cheng D. Chesworth R. Bhatia S. Garner B. Kim W. S.. (2012). Role of Abca7 in mouse behaviours relevant to neurodegenerative diseases. PLoS One 7:e45959. 10.1371/journal.pone.0045959 23029339 PMC3454356

[B29] Logue M. W. Schu M. Vardarajan B. N. Buros J. Green R. C. Go R. C.. (2011). A comprehensive genetic association study of Alzheimer disease in African Americans. Arch. Neurol. 68, 1569–1579. 10.1001/archneurol.2011.646 22159054 PMC3356921

[B30] Ma F.-C. Zong Y. Wang H.-F. Li J.-Q. Cao X.-P. Tan L.. (2018). ABCA7 genotype altered Aβ levels in cerebrospinal fluid in Alzheimer’s disease without dementia. Ann. Transl. Med. 6:437. 10.21037/atm.2018.07.04 30596067 PMC6281527

[B31] Maass A. Düzel S. Goerke M. Becke A. Sobieray U. Neumann K.. (2015). Vascular hippocampal plasticity after aerobic exercise in older adults. Mol. Psychiatry 20, 585–593. 10.1038/mp.2014.114 25311366

[B32] Maesako M. Uemura K. Kubota M. Kuzuya A. Sasaki K. Hayashida N.. (2012). Exercise is more effective than diet control in preventing high fat diet-induced β-amyloid deposition and memory deficit in amyloid precursor protein transgenic mice♦. J. Biol. Chem. 287, 23024–23033. 10.1074/jbc.M112.367011 22563077 PMC3391129

[B33] McGavin C. R. Artvinli M. Naoe H. McHardy G. J. (1978). Dyspnoea, disability and distance walked: Comparison of estimates of exercise performance in respiratory disease. Br. Med. J. 2, 241–243. 10.1136/bmj.2.6132.241 678885 PMC1606354

[B34] McGavin C. R. Gupta S. P. McHardy G. J. (1976). Twelve-minute walking test for assessing disability in chronic bronchitis. Br. Med. J. 1, 822–823. 10.1136/bmj.1.6013.822 1260350 PMC1639415

[B35] Michaelson D. M. (2014). APOE ε4: The most prevalent yet understudied risk factor for Alzheimer’s disease. Alzheimers Dement. 10, 861–868. 10.1016/j.jalz.2014.06.015 25217293

[B36] Myers C. E. Kluger A. Golomb J. Ferris S. de Leon M. J. Schnirman G.. (2002). Hippocampal atrophy disrupts transfer generalization in nondemented elderly. J. Geriatr. Psychiatry Neurol. 15, 82–90. 10.1177/089198870201500206 12083598

[B37] Myers C. E. Kluger A. Golomb J. Gluck M. A. Ferris S. (2008). Learning and generalization tasks predict short-term cognitive outcome in nondemented elderly. J. Geriatr. Psychiatry Neurol. 21, 93–103. 10.1177/0891988708316858 18474718

[B38] Naj A. C. Jun G. Beecham G. W. Wang L.-S. Vardarajan B. N. Buros J.. (2011). Common variants at MS4A4/MS4A6E, CD2AP, CD33 and EPHA1 are associated with late-onset Alzheimer’s disease. Nat. Genet. 43, 436–441. 10.1038/ng.801 21460841 PMC3090745

[B100] Noonan V. Dean E. (2000). Exercise testing: clinical application and interpretation. Phys. Ther. 80, 782–807. 10.1093/ptj/80.8.782 10911416

[B39] N’songo A. Carrasquillo M. M. Wang X. Burgess J. D. Nguyen T. Asmann Y. W.. (2017). African American exome sequencing identifies potential risk variants at Alzheimer disease loci. Neurol. Genet. 3:e141. 10.1212/nxg.0000000000000141 28480329 PMC5406839

[B40] Omoumi A. Fok A. Greenwood T. Sadovnick A. D. Feldman H. H. Hsiung G.-Y. R. (2014). Evaluation of late-onset Alzheimer disease genetic susceptibility risks in a Canadian population. Neurobiol. Aging 35, 936.e5–936.e12. 10.1016/j.neurobiolaging.2013.09.025 24176626

[B41] Pereira A. C. Huddleston D. E. Brickman A. M. Sosunov A. A. Hen R. McKhann G. M.. (2007). An in vivo correlate of exercise-induced neurogenesis in the adult dentate gyrus. Proc. Natl. Acad. Sci. U S A 104, 5638–5643. 10.1073/pnas.0611721104 17374720 PMC1838482

[B42] Podewils L. J. Guallar E. Kuller L. H. Fried L. P. Lopez O. L. Carlson M.. (2005). Physical activity, APOE genotype and dementia risk: findings from the cardiovascular health cognition study. Am. J. Epidemiol. 161, 639–651. 10.1093/aje/kwi092 15781953

[B43] Pooler A. M. Polydoro M. Maury E. A. Nicholls S. B. Reddy S. M. Wegmann S.. (2015). Amyloid accelerates tau propagation and toxicity in a model of early Alzheimer’s disease. Acta Neuropathol. Commun. 3:14. 10.1186/s40478-015-0199-x 25853174 PMC4371800

[B44] Ramirez L. M. Goukasian N. Porat S. Hwang K. S. Eastman J. A. Hurtz S.. (2016). Common variants in ABCA7 and MS4A6A are associated with cortical and hippocampal atrophy. Neurobiol. Aging 39, 82–89. 10.1016/j.neurobiolaging.2015.10.037 26923404

[B45] Rehfeld K. Müller P. Aye N. Schmicker M. Dordevic M. Kaufmann J.. (2017). Dancing or fitness sport? the effects of two training programs on hippocampal plasticity and balance abilities in healthy seniors. Front. Hum. Neurosci. 11:305. 10.3389/fnhum.2017.00305 28674488 PMC5475381

[B46] Reitz C. Honig L. Vonsattel J. P. Tang M.-X. Mayeux R. (2009). Memory performance is related to amyloid and tau pathology in the hippocampus. J. Neurol. Neurosurg. Psychiatry 80, 715–721. 10.1136/jnnp.2008.154146 19258354 PMC2785022

[B47] Reitz C. Jun G. Naj A. Rajbhandary R. Vardarajan B. N. Wang L.-S.. (2013). Variants in the ATP-binding cassette transporter (ABCA7), apolipoprotein E ε4 and the risk of late-onset Alzheimer disease in African Americans. Jama 309, 1483–1492. 10.1001/jama.2013.2973 23571587 PMC3667653

[B48] Ross R. M. Murthy J. N. Wollak I. D. Jackson A. S. (2010). The six minute walk test accurately estimates mean peak oxygen uptake. BMC Pulm. Med. 10:31. 10.1186/1471-2466-10-31 20504351 PMC2882364

[B49] Rovio S. Kåreholt I. Helkala E.-L. Viitanen M. Winblad B. Tuomilehto J.. (2005). Leisure-time physical activity at midlife and the risk of dementia and Alzheimer’s disease. Lancet Neurol. 4, 705–711. 10.1016/S1474-4422(05)70198-8 16239176

[B50] Schuit A. J. Feskens E. J. M. Launer L. J. Kromhout D. (2001). Physical activity and cognitive decline, the role of the apolipoprotein e4 allele. Med. Sci. Sports Exerc. 33, 772–777. 10.1097/00005768-200105000-00015 11323547

[B51] Schultz S. A. Boots E. A. Darst B. F. Zetterberg H. Blennow K. Edwards D. F.. (2017). Cardiorespiratory fitness alters the influence of a polygenic risk score on biomarkers of AD. Neurology 88, 1650–1658. 10.1212/wnl.0000000000003862 28341646 PMC5405766

[B52] Shulman J. M. Chen K. Keenan B. T. Chibnik L. B. Fleisher A. Thiyyagura P.. (2013). Genetic susceptibility for Alzheimer disease neuritic plaque pathology. JAMA Neurol. 70, 1150–1157. 10.1001/jamaneurol.2013.2815 23836404 PMC3773291

[B53] Sinha N. Reagh Z. M. Tustison N. J. Berg C. N. Shaw A. Myers C. E.. (2018). ABCA7 risk variant in healthy older african americans is associated with a functionally isolated entorhinal cortex mediating deficient generalization of prior discrimination training. Hippocampus [Epub ahead of print]. 10.1002/hipo.23042 30318785 PMC6462424

[B54] Smith J. C. Nielson K. A. Woodard J. L. Seidenberg M. Durgerian S. Antuono P.. (2011). Interactive effects of physical activity and APOE-ε4 on BOLD semantic memory activation in healthy elders. Neuroimage 54, 635–644. 10.1016/j.neuroimage.2010.07.070 20691792 PMC2962671

[B55] Szabo A. N. McAuley E. Erickson K. I. Voss M. Prakash R. S. Mailey E. L.. (2011). Cardiorespiratory fitness, hippocampal volume and frequency of forgetting in older adults. Neuropsychology 25, 545–553. 10.1037/a0022733 21500917 PMC3140615

[B56] Tang M.-X. Cross P. Andrews H. Jacobs D. M. Small S. Bell K.. (2001). Incidence of AD in african-americans, cribbean hispanics and caucasians in northern manhattan. Neurology 56, 49–56. 10.1212/wnl.56.1.49 11148235

[B101] Taylor H. L. Buskirk E. Henschel A. (1995). Maximal oxygen intake as an objective measure of cardio-respiratory performance. J. Appl. Physiol. 8, 73–80. 10.1152/jappl.1955.8.1.73 13242493

[B57] Van Praag H. (2008). Neurogenesis and exercise: past and future directions. Neuromolecular Med. 10, 128–140. 10.1007/s12017-008-8028-z 18286389

[B58] Van Praag H. Shubert T. Zhao C. Gage F. H. (2005). Exercise enhances learning and hippocampal neurogenesis in aged mice. J. Neurosci. 25, 8680–8685. 10.1523/jneurosci.1731-05.2005 16177036 PMC1360197

[B59] Vasquez J. B. Fardo D. W. Estus S. (2013). ABCA7 expression is associated with Alzheimer’s disease polymorphism and disease status. Neurosci. Lett. 556, 58–62. 10.1016/j.neulet.2013.09.058 24141082 PMC3863933

[B60] Yaffe K. Barnes D. Nevitt M. Lui L.-Y. Covinsky K. (2001). A prospective study of physical activity and cognitive decline in elderly women: women who walk. Arch. Intern. Med. 161, 1703–1708. 10.1001/archinte.161.14.1703 11485502

[B61] Zhao Q.-F. Yu J.-T. Tan M.-S. Tan L. (2015). ABCA7 in Alzheimer’s disease. Mol. Neurobiol. 51, 1008–1016. 10.1007/s12035-014-8759-9 24878767

[B62] Zhao X.-S. Wu Q. Peng J. Pan L.-H. Ren Z. Liu H.-T.. (2017). Hyperlipidemia-induced apoptosis of hippocampal neurons in apoE(−/−) mice may be associated with increased PCSK9 expression. Mol. Med. Rep. 15, 712–718. 10.3892/mmr.2016.6055 28000893 PMC5364825

